# ATM inhibition overcomes resistance to histone deacetylase inhibitor due to p21 induction and cell cycle arrest

**DOI:** 10.18632/oncotarget.27723

**Published:** 2020-09-15

**Authors:** Luigi Scotto, Xavier J. Serrano, Kelly Zullo, Cristina Kinahan, Changchun Deng, Ahmed Sawas, Susan Bates, Owen A. O’Connor

**Affiliations:** ^1^Division of Experimental Therapeutics, Department of Medicine, Columbia University Medical Center, New York, NY, USA; ^2^Center for Lymphoid Malignancies, Department of Medicine, Columbia University Medical Center, New York, NY, USA; ^3^Division of Hematology and Oncology, Department of Medicine, Columbia University Medical Center, New York, NY, USA

**Keywords:** lymphoma, HDAC inhibitor, ATM inhibitor, p21, cell cycle

## Abstract

The antiproliferative effect induced by histone deactylase inhibitors (HDACi) is associated with the up-regulated expression of the cyclin-dependent kinase inhibitor p21. Paradoxically, the increased expression of p21 correlates with a reduced cell killing to the drug. The direct targeting of p21 is not feasible. An alternate approach could selectively target factors upstream or downstream of p21 that affect one or more specific aspects of p21 function. HDAC inhibitors appear to activate p21 expression via ataxia telangiectasia mutated (ATM) activity. KU60019, a specific ATM inhibitor, has shown to decrease the p21 protein levels in a concentration dependent manner. We explored the potential synergistic interaction of the ATM inhibitor with romidepsin, given the potential complementary impact around p21. A synergistic cytotoxic effect was observed in all lymphoma cell lines examined when the HDACi was combined with KU60019. The increase in apoptosis correlates with decreased expression of p21 due to the ATM inhibitor. KU60019 decreased expression of the cyclin-dependent kinase inhibitor at the transcriptional level, compromising the ability of HDACi to induce p21 and cell cycle arrest and ultimately facilitating a shift toward the apoptotic phase. Central to the increased apoptosis observed when romidepsin is combined with KU60019 is the reduced expression of p21 and the absence of a G2/M cell cycle arrest that would be exploited by the tumor cells to evade the cytotoxic effect of the HDAC inhibitor. We believe this strategy may offer a promising way to identify rational combinations for HDACi directed therapy, improving their activity in malignant disease.

## INTRODUCTION

HDAC inhibitors (HDACi) have emerged as valuable drugs in the treatment of select lymphomas and synergize with a diverse range of pharmacological and biological agents [1]. A common feature of many HDAC inhibitors involves induction of cell cycle arrest which is explained in part by the induction of CDKN1A (encoding p21WAF1/CIP1) [2, 3]. Ironically, up-regulation of p21 has been shown to reduce the sensitivity to killing by HDAC inhibitors [4]. The observation leads to the following hypothesis: if induction of p21 compromises the efficacy of HDAC inhibitors, then strategies to mitigate HDAC inhibitor induced p21 expression could lead to promising synergistic combinations. p21 plays a complex role in cancer, displaying functions identified as both tumor suppressor and tumor promoting; and as anti- or pro-apoptotic, each depending on the cellular context [5, 6]. The direct targeting of p21 is probably not feasible given the strong evidence for the tumor-suppressor functions of p21 as a regulator of genomic stability, and its role in control of senescence in normal cells. Selectively targeting factors upstream or downstream of p21 function may affect a more specific aspect of p21 control. Ataxia telangiectasia (A-T) is a disorder caused by mutations in the ataxia telangiectasia mutated (ATM) gene which controls cell division and DNA repair [7]. Induction of p21 by HDAC inhibitors is compromised in A-T cells given that ATM activity is essential for HDAC inhibitor-induced p21 expression [8]. Collectively, these observations have led to the following hypothesis: If ATM activity is necessary for HDAC inhibitor mediated p21 induction, then selective ATM inhibitors (KU60019) could mitigate the HDAC induced p21 expression and potentiate its cytotoxic effect. We report herein that romidepsin and KU60019 are highly synergistic in *in vitro* models of lymphoma. The increased cell death correlates with increased activation of programmed cell death proteins (Caspase 3 and Caspase 8) and a substantial decrease in the expression of anti-apoptotic genes (BCL-2 and BCL-XL). The increase in apoptosis seen with KU60019 is associated with a coincident absence of p21 induction by romidepsin. The ATM inhibitor nullifies HDAC induction of p21 expression resulting in a synergistic interaction. KU60019 reduces p21 expression at the transcriptional level and antagonizes romidepsin transcriptional induction of p21. In both instances the result is a markedly down-regulation of p21 expression at the protein level.

## RESULTS

### Romidepsin influences expression of proteins involved in cell cycle regulation and induces a G2/M arrest in mantle cell lymphoma

**Figure 1:**
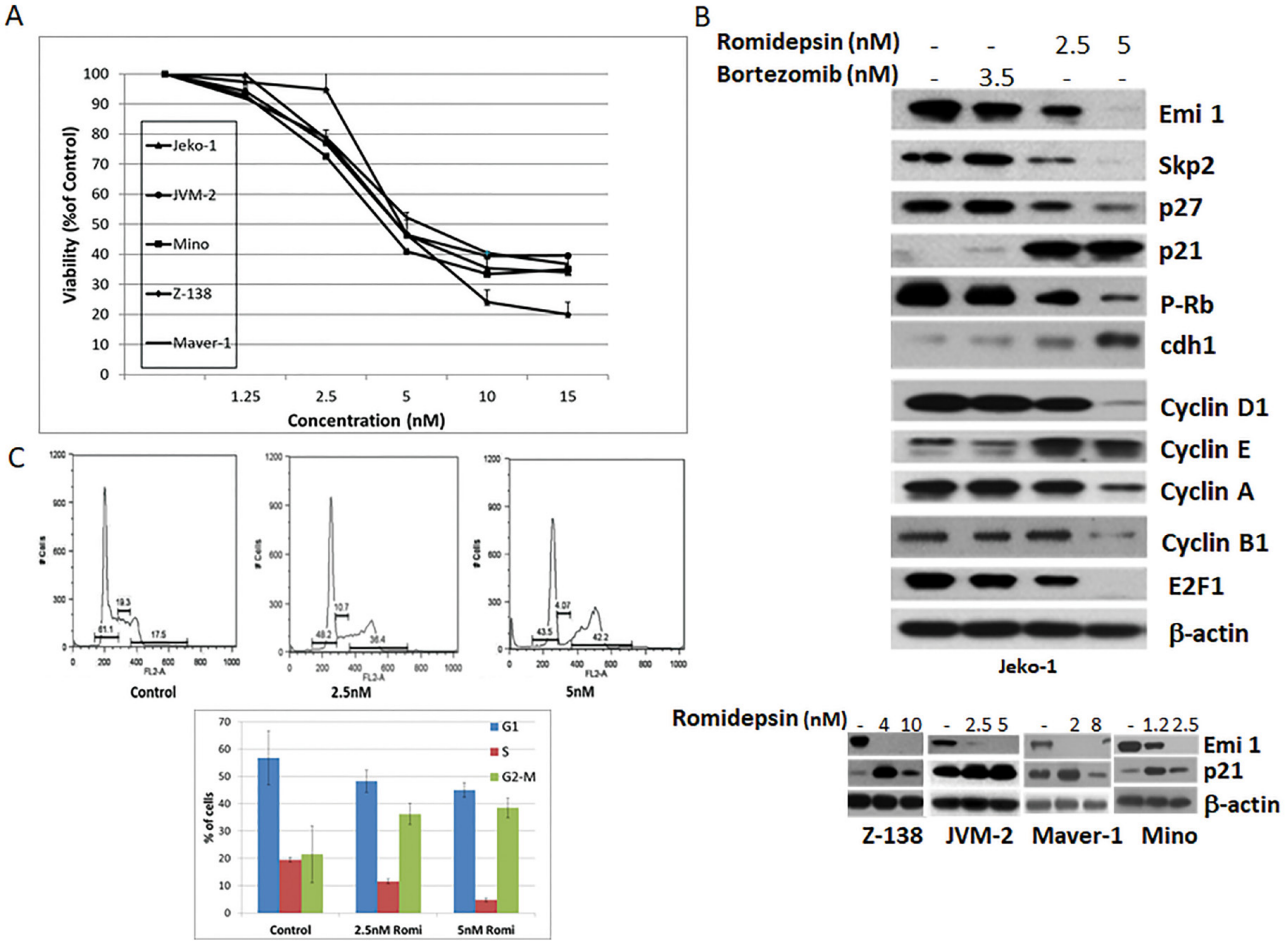
Romidepsin affects expression of proteins involved in cell cycle regulation in MCL. (**A**) Growth inhibition curves were generated for a panel of MCL cell lines following 24 hours treatment with increased concentrations of romidepsin. (**B**) Protein expression analysis of Jeko-1 cells following 24 hours treatment with bortezomib or romidepsin (upper panel). Emi 1 and p21 protein expression in a panel of MCL cell lines following 24 hours romidepsin exposure (lower panel). (**C**) Cell cycle analysis of Jeko-1 cells following 24 hours romidepsin treatment. Error bars represent SD.

*In vitro* romidepsin exhibited concentration-dependent cytotoxicity against a panel of MCL cell lines with a half maximal inhibitory concentration (IC50) in the 2.5–5.0 nmol/L range after 24 hours. (Figure 1A). Protein expression analysis of Jeko-1 cells exposed to romidepsin produced a decrease in E2F1 and Emi1 protein levels when compared to the untreated cells (Figure 1B, upper panel). The decrease in Emi1 expression was associated with a concomitant decrease in expression of the E3 ubiquitin protein ligase Skp2, known to mediate the ubiquitination and subsequent proteasome mediated degradation of phosphorylated cyclin-dependent kinase inhibitor p27. While no increase in p27 expression was observed upon addition of the HDACi, the expression of p21 was markedly up-regulated. Simultaneous accumulation of p27 and p21 was observed in Jeko-1 cells exposed for 24 hours to 3.5 nmol/L bortezomib (Figure 1B, upper panel). The decrease in Emi1 and increase in p21 protein levels were also observed in four other romidepsin treated mantle cell lymphoma lines as noted (Figure 1B, lower panel). The expression of other key regulators involved in the control of cell cycle was also affected by romidepsin, including reduction in Cyclins D1, A, and B1, and an increase in expression of Cyclin E and Cdh1 (APC) (Figure 1B, upper panel).

The induced expression of p21 is considered to be a contributing factor to the G1/S and G2/M cell cycle arrest [9, 10]. p21 inhibits cell cycle progression primarily through the inhibition of CDK2 activity, which is required for the phosphorylation of RB and the consequent release and activation of E2F-dependent gene expression [11]. Fluorescence-activated cell sorting (FACS) analysis of Jeko-1 cells exposed to romidepsin revealed an induced G2/M arrest. Romidepsin induced cell cycle arrest in 36.2 and 38.4% of Jeko-1 cells when compared to 21% of untreated cells (Figure 1C), with a concurrent decrease in G1 and S phase cell populations.

### The KU60019 ATM inhibitor and romidepsin are highly synergistic in *in vitro* models of MCL

Up-regulation of p21 reduces sensitivity to killing by HDACi [4]. Ju et al. (2003) [8] reported that p21 induction by HDAC inhibitors is defective in Ataxia telangiectasia (AT) cells, supporting the idea that ATM activity is necessary for HDAC inhibitor-induced p21 expression. We reasoned that inhibiting ATM activity (with KU60019) in presence of romidepsin would interfere with the HDACi induced expression of p21 and would increase its cytotoxic effects.

**Figure 2:**
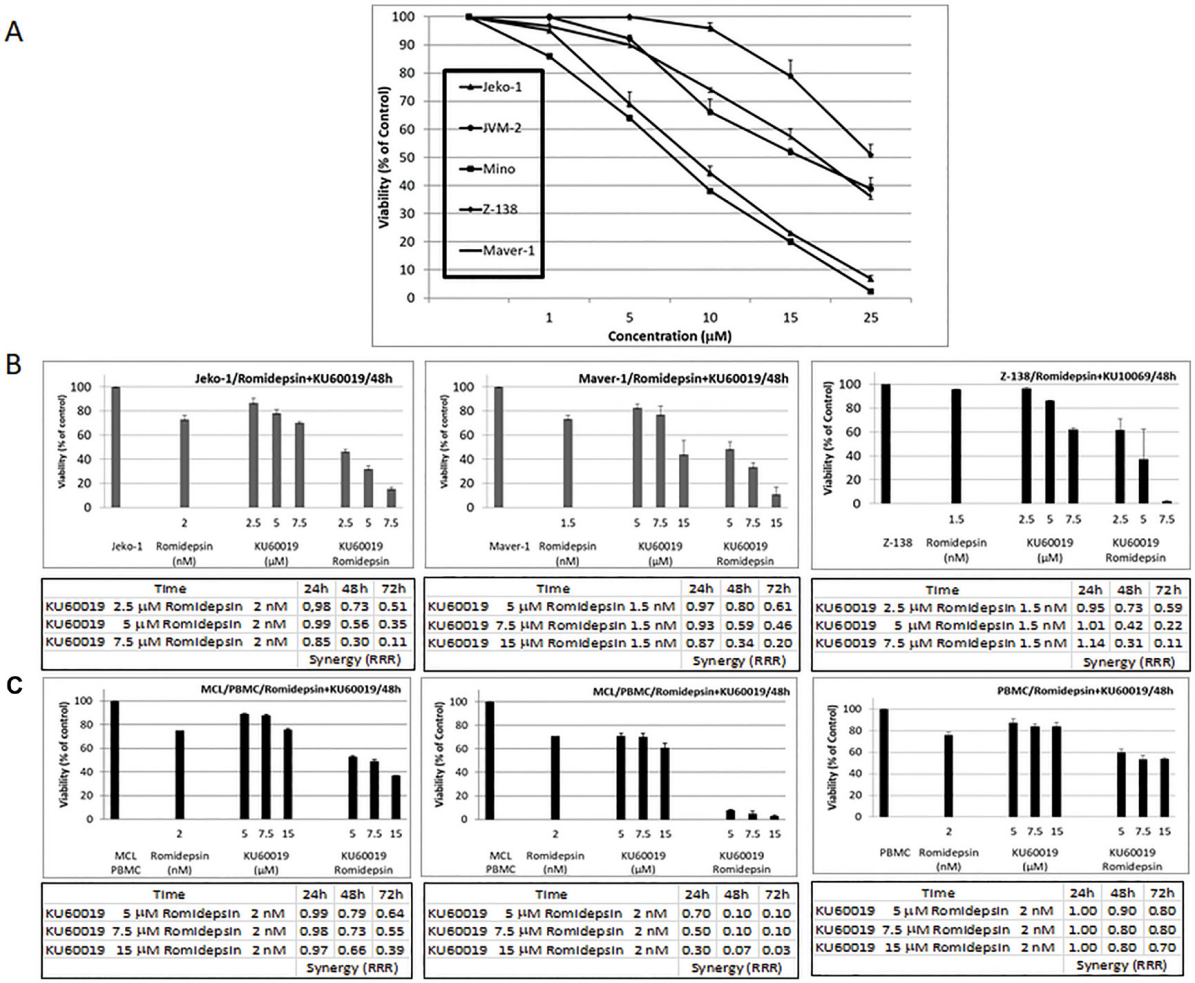
The ATM inhibitor KU60019 and Romidepsin are highly synergistic in *in vitro* models of MCL. (**A**) Growth inhibition curves were generated for the panel of MCL cell lines following 48 hours of KU60019 exposure. (**B**) Cytotoxicity effects observed at 48 hours of exposure to single agents and combination for Jeko-1, Maver-1, and Z-138 MCL cell lines. (**C**) Cytotoxicity effects observed at 48 hours of exposure to single agents and combination for PBMC isolated from a patient diagnosed with classic MCL, blastoid variant of MCL and a healthy donor. Also shown are RRR values at 24, 48, and 72 hours of exposure to romidepsin in combination with KU60019. Error bars represent SD.

KU60019 exhibited concentration-dependent cytotoxicity against a panel of MCL cell lines with an IC50 between 10 to 20 μmol/L (Figure 2A). Synergy analyses were performed using Jeko-1, Maver-1, and Z-138 cells treated with different concentrations of romidepsin corresponding to the IC10-20 in combination with KU60019. A synergistic effect was observed in all cell lines when the HDACi was combined with KU60019 throughout the range of concentrations explored (Figure 2B). The relative risk ratio (RRR) analysis revealed a strong synergism at 48 and 72 hours in all combinations in all three cell lines with RRR values at 72 hours ranging between 0.5 and 0.1. The cell viability data following treatment with the single agents or combinations at 48 hours are shown in Figures 2B. Synergy analyses were also performed using peripheral blood mononuclear cells (PBMC) isolated from two patients diagnosed with a classic MCL and a blastoid variant of MCL, respectively. A strong synergistic effect was observed with the combination (Figure 2C). The RRR values at 72 hours ranged between 0.6 and 0.03. Cell viability data after treatment with the single agents showed less than 10% or 40% viability when the HDACI was combined with KU60019. On the contrary, RRR values between 0.8 and 0.7 and 60% viability were observed for PBMC isolated from two healthy donors throughout the same range of concentrations (Figure 2C and Supplementary Figure 1). These data suggest a favorable therapeutic window with less toxicity against normal lymphocytes.

### Combination of romidepsin plus KU60019 enhances apoptosis in MCL lines

**Figure 3:**
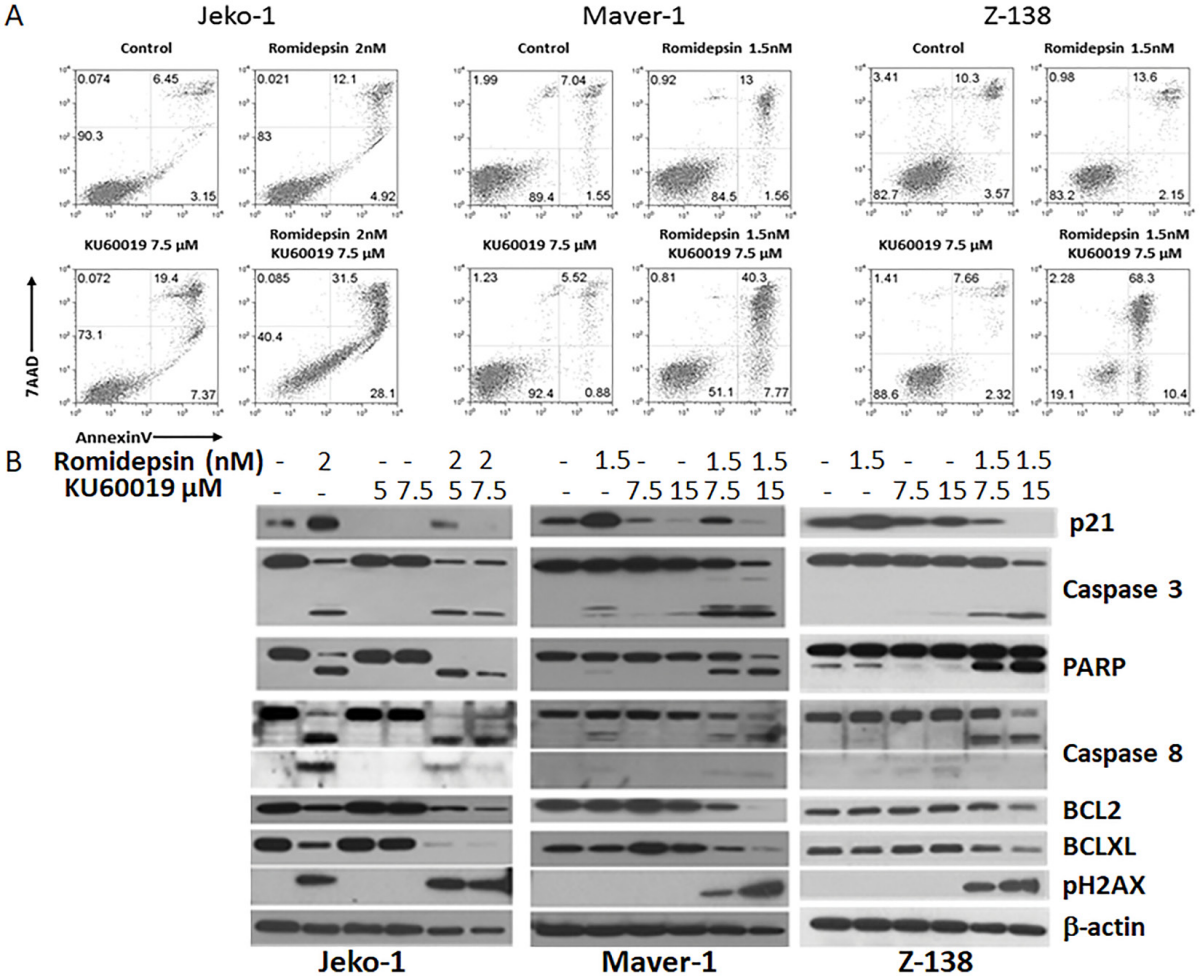
Romidepsin plus KU60019 enhances apoptosis in MCL. (**A**) Cytofluorimetric detection of apoptosis in Jeko-1, Maver-1 and Z-138 MCL cell lines after 48 hours of exposure to single agents and combination. (**B**) Expression analysis of p21, pH2AX, pro- and anti-apoptotic proteins in Jeko-1, Maver-1, and Z-138 MCL cell lines after 48 hours of exposure to single agents and combination.

Cytofluorimetric analysis of the MCL cells treated with romidepsin and/or KU60019 showed a robust increase in apoptotic cells when the effect of single agents was compared to romidepsin plus KU60019 combination (Figure 3A). Protein expression analysis of Jeko-1, Maver-1, and Z-138 treated with single agents or combinations for 48 hours revealed changes in a host of proteins known to be involved in cell cycle control and apoptosis. First, there was no increased p21 protein expression associated with romidepsin when all three cell lines were treated concurrently with KU60019. Second, there was a marked decrease in p21 protein compared to the untreated cells as a function of increasing ATM inhibitor concentration (Figure 3A and Supplementary Figure 2), suggesting a dominant negative effect of KU60019 over the HDACi on the expression of the cyclin dependent kinase inhibitor. Thirdly, there was increased activation of the programmed cell death proteins with the combination, resulting in an increased cleavage of Poly(ADP-ribose)-polymerase (PARP). Finally the abundance of the anti-apoptotic proteins Bcl-XL and BCL-2 showed a significant decrease after treatment with the combination compared with their abundance following exposure to either of the single agents (Figure 3B). These data validated the hypothesis: interfering with the HDACi induced expression of p21 by inhibiting the catalytic activity of its upstream effector would translate into increased sensitivity to the HDACi.

**Figure 4:**
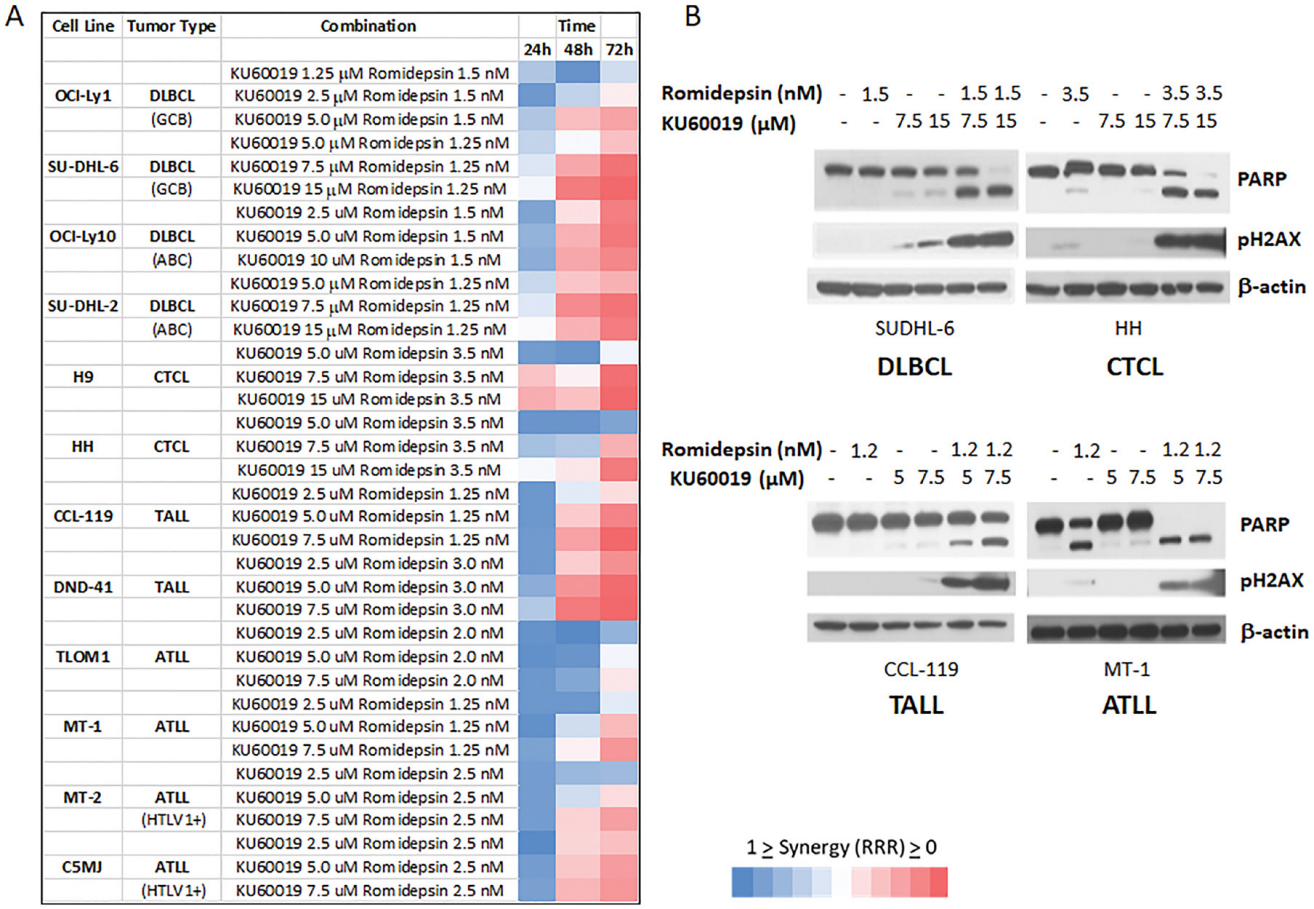
The ATM inhibitor KU60019 and Romidepsin are highly synergistic in *in vitro* models of DLBCL, CTCL, TALL, ATLL. (**A**) Romidepsin and KU60019 were used as single agents and combination to assess their cytotoxicity on a panel of human derived lymphoma cell lines. The relative risk ratio values at 24, 48, and 72 h of exposure are shown. RRR values < 1 represent the synergistic effect of the 2 drugs; values equal to 1 indicate the mean additive effect of the drugs; and values > 1 represent an antagonistic effect. (**B**) Protein expression of PARP and pH2AX after 48 hours of exposure to single agents and combination. One lymphoma cell line per tumor type is shown.

Considering the synergistic effect observed when romidepsin was combined with KU60019 in the *in vitro* models of MCL a panel of B- and T-cell derived lymphoma lines that included four diffuse large B-cell lymphoma (DLBCL), two CTCL, four adult T- cell leukemia/lymphoma (ATLL), and two acute T-lymphoblastic leukemia/lymphoma (TALL) were studied. A strong synergistic effect (RRR < 0.5) was observed for all cell lines (Figure 4A). Western analysis of PARP and phosphorylated H2AX expression levels in one representative cell line for each lymphoma subtype confirmed that addition of the ATM inhibitor to romidepsin increases the apoptotic effects of the HDACi (Figure 4B).

### KU60019 affects the ability of romidepsin to induce expression of p21

The results of the protein expression analysis of MCL lines untreated and treated with the single agents and combination suggested that upon addition of the HDACi, Jeko-1, Maver-1, and Z-138 cells undergo cell cycle arrest and apoptosis. However in combination with KU60019 the increase in PARP cleavage and the accumulation of phosphorylated H2AX, critical for DNA degradation, suggests a preferential shift toward apoptosis (Figure 3A and 3B) as result of the ATM inhibition. By compromising the induced expression of p21 and the G2/M cell cycle arrest, KU60019 appears to potentiate romidepsins’ cytotoxic effect. The ATM inhibition mediated by KU60019 over-rides the protective effect seen with p21 induction following romidepsin.

**Figure 5:**
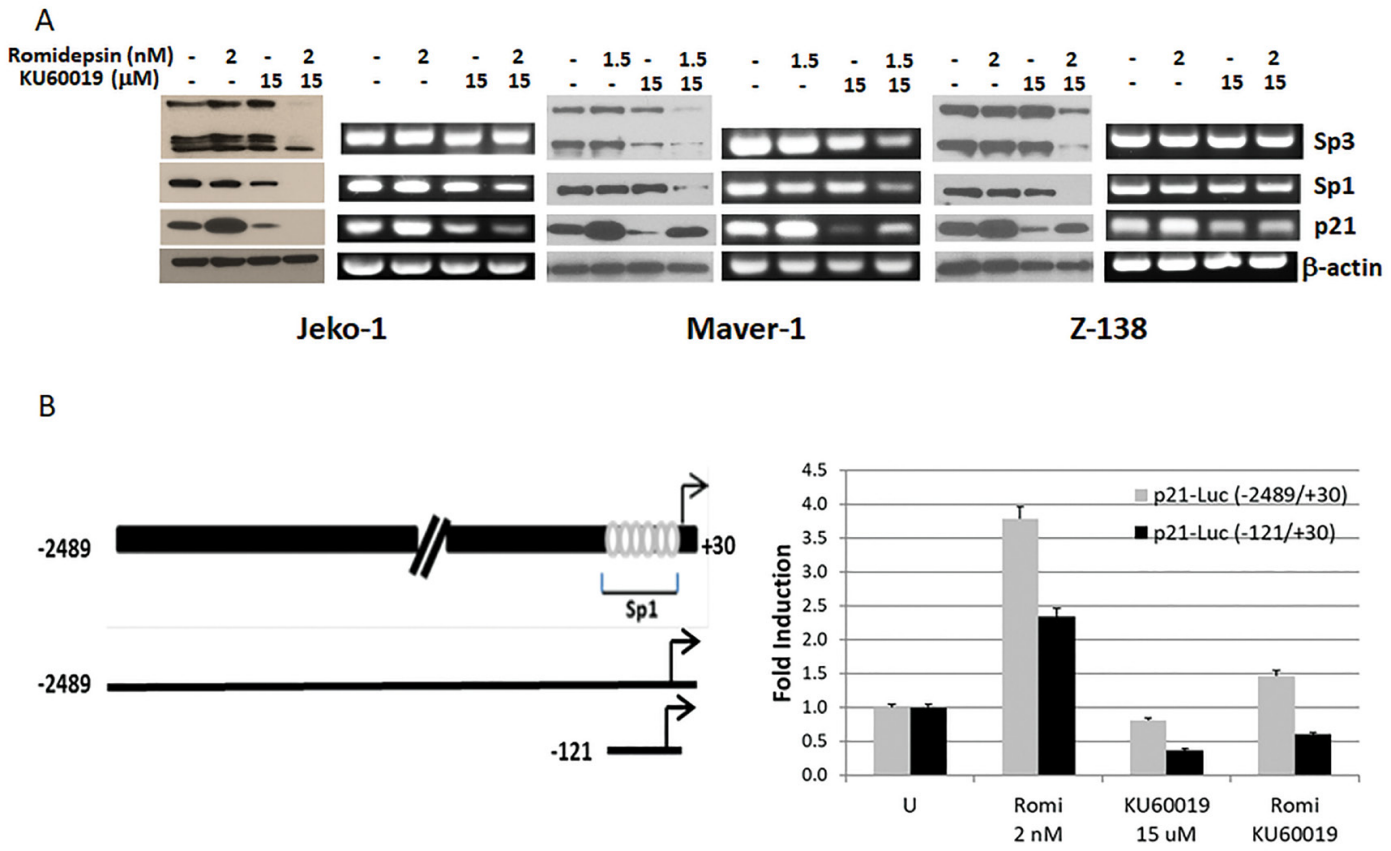
KU60019 affects the ability of romidepsin to induce expression of p21. (**A**) Protein and RT-PCR expression analysis of p21 and transcription factors Sp1, Sp3 in Jeko-1, Maver-1, and Z-138 cells after 24 hours of exposure to single agent and combination. (**B**) Transient transcription analysis of p21 promoter luciferase gene fusion constructs. Reporter plasmids were transiently transfected in Jeko-1 cells and luciferase activity was measured after 24 hours exposure to single agents and combination. Schematic representation of p21 promoter region with depicted Sp1 binding sites and cloned DNA regions. Sp3 primers were designed so to identify both alternative transcripts. Luciferase basal activity of the two constructs is arbitrarily set to 1.0. Error bars represent SD.

Expression of p21 is controlled at the transcriptional level by both p53-dependent and -independent mechanisms [5]. HDACi induced expression of p21 is p53 independent [12] and the transcription factors Sp1 and Sp3 are believed to mediate HDACi induced p21 transcription through their interaction with the p21 promoter [13]. To gain insights into the molecular mechanisms involved in KU60019 interference of p21 induced expression by romidepsin, protein and transcript levels of p21, Sp1 and Sp3 were determined in Jeko-1, Maver-1, and Z-138 cells following 24 hours treatment with the single agents and combination (Figure 5A). Increase of p21 protein and transcript levels were observed in all cell lines following 24 hours exposure to romidepsin when compared to untreated cells. In contrast, a decrease in p21 protein and transcript levels was detected following exposure to KU60019 when compared to untreated cells. However, no change in protein or transcript levels for Sp1 and Sp3 transcription factors were observed. When romidepsin and KU60019 are combined a decreased expression at the transcriptional and/or translational level of SP1, Sp3, and p21 was observed,

To confirm that KU60019 affects the ability of romidepsin to induce p21 at transcriptional level we used p21 promoter deletion-luciferase reporters, with the longest construct extending to 2489 bp upstream of the transcription initiation site in the human p21 gene. The deletion constructs contained 2489 bp and 121 bp of p21 promoter region, respectively (Figure 5B), cloned upstream of the luciferase gene in the pGL3 vector. The sequence of the p21 promoter between −121 and +20 bp contains six Sp1 binding sites that could mediate super-activation of the p21 promoter via a Sp1-dependent mechanism [14]. Jeko-1 cells, evenly transfected with the luciferase reporters, were exposed for 24 hours to the single agents and combination to assess the effect of romidepsin, KU60019 and combination on luciferase expression via luciferase activity. A 3.75- and 2.25-fold increase in luciferase activity was observed in presence of romidepsin with each construct but not in the presence of the ATM inhibitor or the combination. The results support the hypothesis that KU60019 affects the ability of romidepsin to induce the expression of the p21 gene at the transcriptional level and that other transcription factors besides SP1 (i.e., SP3) play a role in the p53-independent expression of p21.

### Inhibition of ATM mediated activation of the G2/M checkpoint by KU60019 facilitates mitotic exit

**Figure 6:**
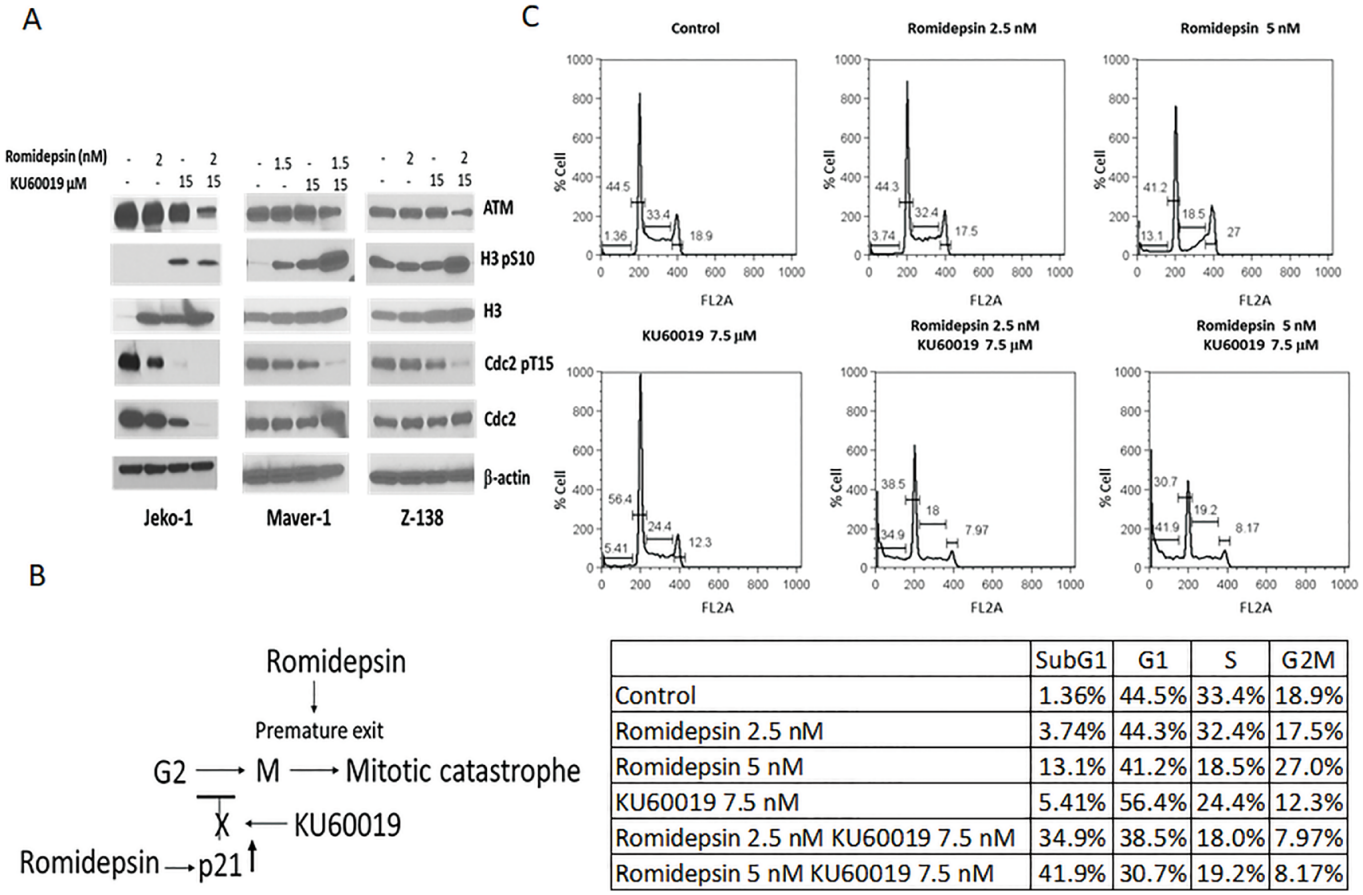
Inhibition of ATM mediated activation of the G2/M checkpoint by KU60019 facilitates mitotic exit. (**A**) Protein expression analysis of ATM, H3, and CDC2 in Jeko-1, Maver-1, and Z-138 cells exposed 24 hours to romidepsin and KU60019 as single agents and combination (**B**) KU60019 inhibit HDACi mediated p21 transcriptional induction facilitating G2 to M transition. Premature mitotic exit induced by romidepsin promote apoptosis (**C**) Cell cycle analysis of Jeko-1 cells following 24 hours exposure to romidepsin and KU60019 as single agents and combination.

Phosphorylation at a highly conserved serine residue (Ser-10) in the histone H3 tail is coincident with mitotic chromosome condensation [15, 16]. Phosphorylation of cdc2 at tyrosine residue (Tyr-15) indicates entry into mitosis [17]. Histone H3-serine-10 phosphorylation as well as reduced cdc2 phosphorylation have been used previously as mitotic markers for transition from G2 to M phase of cell cycle. Western blot analysis of Jeko-1, Maver-1, and Z-138 exposed to single agents or combination reveals an increase histone H3-serine-10 phosphorylation and correlated cdc2-Tyr-15 dephosporylation in the presence of KU60019 that persist or increase when cells are exposed to the combination, suggesting the release of a potential checkpoint in early mitosis (Figure 6A).

HDACi kills cancer cells by inducing aberrant mitotis and premature mitotic exit, which result in the rapid onset of apoptosis. Blocking mitotic exit in the presence of the HDACi has been reported to inhibit apoptosis [18]. Cell cycle analysis of Jeko-1 cells exposed to single agents and the combination confirmed that KU60019 compromises the ability of romidepsin to induce a G2/M cell cycle arrest in Jeko-1 cells allowing them to proceed into mitosis (Figure 6B). Ultimately, KU60019 contributes to tumor cell killing by blocking the HADCi mediated p21 transcriptional induction facilitating the G2 to M transition while romidepsin induce aberrant mitosis and premature mitotic exit (Figure 6C).

### The combination of romidepsin and KU60019 is synergistic in a xenograft model of MCL

**Figure 7:**
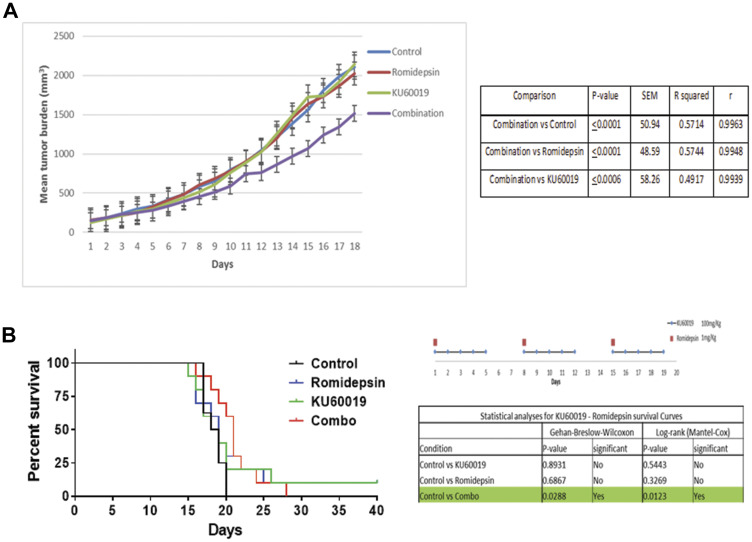
The combination of romidepsin and KU60019 is synergistic in a xenograft model of MCL. (**A**) The *in vivo* therapeutic efficacy of romidepsin and KU60019 as single agents and combination was evaluated using a SCID Beige xenograft mouse model of MCL. The effectiveness of romidepsin in combination with KU60019 was evaluated in a 3 week cycle administration. Romidepsin was administered weekly at days 1, 8, and 15. KU60019 was given at days 1, 2, 3, 4, and 5 of each week. The data are expressed as average tumor volume (mm^3^) per group as a function of time. A two-tailed *t* test confirmed that combination was statistically superior to both the single agents and the control in inhibiting tumor growth (0.001 < *P* < 0.05). (**B**) Statistical analysis of survival following the 3 weeks administration cycle. When compared to control, only the romidepsin plus KU60019 combination was statistically significant. *P*-value, standard error of the mean (SEM), coefficient of determination (R squared), and coefficient of correlation (*r*) of comparisons.

The *in vivo* efficacy of romidepsin (1 mg/kg) combined with KU60019 (100 mg/kg) was evaluated in a xenograft mouse model of MCL using the Z-138 cell line. Figure 7A shows the median tumor volume in each cohort over time during treatment displaying a pattern of statistically significant growth delay favoring the combination over single agents and vehicle control. Statistical analysis revealed that the combination of romidepsin plus KU60019 was superior to romidepsin, KU60019 or the vehicle control. Two animals in the romidepsin treated group and one in the KU60019 treated cohort had to be sacrificed because of progressive disease (tumor volume exceeding 2000 mm^3^) before day 18, while all animals in the control group were sacrificed by day 18 (tumor volume exceeding 2000 mm^3^) (Figure 7A). Survival was improved in the cohorts of mice treated with the combination of romidepsin and KU60019 compared to mice treated with single agent alone or vehicle control (Figure 7B). Importantly, a statistical significant improvement in survival was only seen in the combination cohort.

## DISCUSSION

The cyclin-dependent kinase inhibitor p21 was initially identified for its ability to cause cell cycle arrest. Induced upon stress by p53-dependent and p53-independent mechanisms, the cyclin-dependent kinase inhibitor allows the cells to pause during cell cycle and repair DNA damage. Because of its ability to inhibit cell proliferation p21 has been considered a tumor suppressor [19]. Recent analyses of its functions have suggested a dual role for p21 as both a classical tumor suppressor and an oncogene. In particular, the differential effects of p21 on apoptosis and sensitivity to cancer chemotherapy is under study in view of a more rational approach to drug design and therapeutic strategy.

One of the mechanisms by which p21 can prevent apoptosis involves p21-dependent cell cycle arrest. Several anticancer agents interfere with or damage the basic mechanisms of DNA synthesis activating the DNA repair pathway and promoting the induction of the cyclin-dependent kinase inhibitor p21 leading to cell cycle arrest [20]. Resistance to drug treatment, not related to p21 induced cell cycle arrest, has also been associated with up-regulated expression of p21 [21, 22].

The direct targeting of p21 is not feasible due to its role in maintaining genomic stability and allowing DNA repair in normal tissue. An alternate approach to target the unfavorable influences of p21 on cancer cell treatment could selectively target factors upstream or downstream of p21 that affect one or more specific aspects of p21 function. In this context the compromised induction of p21 by HDAC inhibitors in A-T cells and the observation that ATM appears to be necessary for HDAC inhibitor-induced p21 expression [8] has led us to interrogate the potential synergistic effects of ATM inhibition, as one means to augment sensitivity to HDACi. We have used a panel of MCL cell lines and extended our analysis to other lymphoma sub-types including DLBCL, CTCL, ATLL, and TALL. In all scenarios, the combination of the HDAC and ATM inhibitor produced a significant increase in the tumor cell killing when compared to the untreated or single agent treated cells. Moreover the increase in apoptosis was also observed in PBMC isolated from two patients diagnosed with MCL.

The increased synergy was clearly associated with the inability of romidepsin to induce the expression of the cell cycle regulatory protein p21 in the presence of KU60019. The ATM inihibitor, decreased p21 protein expression and in combination with romidepsin interferes with the ability of the HDACi to induce p21 at the transcriptional level.

Inhibition of the catalytic activity of ATM for the treatment of cancer has been a target for antineoplastic based drug discovery. Cancer cells routinely carry mutations in genes affecting the cell cycle and DNA repair [23]. ATM-deficient cells cannot induce checkpoint arrest following DNA damage and the cell is directed to a suicide route by an alternative, p53-independent mechanism [24]. New and more selective ATM inhibitors are in development given the therapeutic effects seen in *in vitro* and *in vivo* model studies and an improved understanding of the multiplicity of critical cellular operations regulated by ATM and ATM dependent pathways.

HDACi are thought to kill tumor cells by driving premature exit from aberrant mitosis and inducing the consequent rapid onset of apoptosis. Blocking mitotic exit in presence of HDACi has been reported to inhibit apoptosis [18, 25]. It is conceivable to think that the G2/M arrest observed in Jeko-1 cells exposed to romidepsin protect them from the cytotoxic effects of the HDACi and assure survival once the drug is removed and hyperacetylation reversed. In this case, by affecting romidepsins’ ability to induce p21 transcriptionally, KU60019 can enable the cells to proceed to mitosis. In view of the recent role described for ATM in the activation of the spindle checkpoint it is plausible that the premature exit from an aberrant mitosis is also enhanced by the ATM catalytic inhibition [26].

It is intuitive that pleotropic drugs like HDACi are likely to have both favorable and unfavorable effects on cell growth and survival. Strategies directed toward understanding how to mitigate the unfavorable influences of the class can lead to improved efficacy in rational combinations. Many examples of drug synergy with HDAC inhibitors have been driven by random efforts in mixing and matching in order to identify possible complementary partners. Obviously, a clear understanding of the molecular pharmacologic features of pleotropic drug classes like HDAC inhibitors can afford unique opportunities to think about logical combinations. Ultimately, these approaches need to be translated to the clinic in order to establish therapeutic merit in the clinic.

## MATERIALS AND METHODS

### Cell lines

Authentication of cell lines was performed by ATCC through short tandem repeat (STR) profiling. All cell lines were tested for mycoplasma and were cultured in RPMI 1640 medium or Iscove modified Dulbecco medium (Invitrogen) containing 10% (v/v) heat inactivated fetal bovine serum (Invitrogen) at 37°C under 5% CO2. Patients and donor venous blood was obtained following informed consent. Venous blood was drawn into sodium heparin and peripheral blood mononuclear cells (PBMC) separated immediately. PBMC were isolated from anticoagulated venous blood by centrifugation over Ficoll Hypaque (GE Healthcare Bio-Sciences).

### Materials

Romidepsin (FK228, Depsipeptide) and KU60019 were purchased from Selleckchem.

### Cytotoxicity assays

For all *in-vitro* assays, cells were counted, incubated, and processed as previously described [27, 28]. Romidepsin and KU0019 were diluted in DMSO to a final concentration of ≤ 0.01%. For combination experiments, the final concentration of all drugs was selected to approximate the IC10-IC30. For all cytotoxicity experiments, Cell-Titer-Glo Reagent (Promega), a Synergy HT Multi-Detection Microplate Reader (Biotek Instruments, Inc.) were used as previously described [28]. Synergistic interactions were measured using the Relative Risk Ratio (RRR) [27, 28].

### Flow cytometry

Cells (3 × 10^5^ cells/mL) were incubated for 24 or 48 hours with romidepsin and KU60019, alone or in combination at concentrations approximating the IC10-IC30. A minimum of 1 × 10^5^ events were acquired for each sample. To quantitate apoptosis, cells were stained with Alex Fluor 488/Annexin V and 7-aminoactinomycin D (7-AAD) (Invitrogen) according to the manufacturer’s instruction. For cell cycle analysis cells were stained with propidium iodide (Invitrogen) according to the manufacturer’s instruction. Flow cytometry was performed on a FACS Calibur System and the data were analyzed with Flowjo 8.8.6 software.

### Western blotting

Western blotting was performed as previously described [28]. Primary antibody anti-Emi1 (Invitrogen), anti-Sp1, -Sp3 (Santa Cruz Biotechnology). All other primary and secondary antibodies (Cell Signaling Technology).

### Semiquantitative reverse-transcribed PCR

Reverse transcription was performed in a 20-μL reaction volume with a total of 2 ug of RNase free DNase-treated RNA using Omniscript RT kit (QIAGEN) and oligo-d(T). Polymerase chain reactions were run in 30 thermal cycles; PCR products were observed by electrophoresis on 2% agarose gel and visualized after staining with ethidium bromide. Primers are listed in Supplementary Table 1.

### Transient transfection

The Jeko-1 cells were transiently transfected using Nucleofector 1 (Amaxa) according to the manufacturer’s protocol. Each transfection was done in triplicate and luciferase activity was measured 24 hours after treatment using the Luciferase Assay System (Promega) according to the manufacturer’s instructions. Normalized values are reported as the mean. Standard deviation (SD) is calculated from three independent transfections.

### Statistical analysis

IC50 (half the maximal inhibitory concentration) for each cell line was calculated using the Calcusyn Version 2.0 software (Biosoft) [29]. Relative risk ratio (RRR) was used as a model for establishing synergy between 2 drugs. RRR is based on calculating the ratio between the actual value and expected value (EV). In the case of 2 cytotoxic compounds EV is calculated by formula: EV = NA × NB/100, where NA represents the percentage of viable cells in the sample treated with drug A and NB represents the percentage of viable cells in the sample treated with drug B. RRR values < 1 represent the synergistic effect of the 2 drugs, values equal to 1 indicate the mean additive effect of the drugs, and values > 1 represent an antagonistic effect [28].

### *In vivo* studies

Five to seven week-old SCID beige mice (Charles River) were injected subcutaneously with Z-138 (5 × 10^6^) cells in the posterior flank. When tumors approached 150 mm^3^ mice were randomized in 4 groups of 8 mice: (1) control group received saline with 10% DMSO; (2) romidepsin (1 mg/Kg intraperitoneally) alone group; (3) KU60019 (100 mg/Kg oral gavage) alone group; (4) combination group (treatment schedule). Romidepsin was administered in PBS; KU60019 in a PEG-400: 45% PBS: 10% DMSO solution. Mice were monitored for 40 days post enrollment. Animals were maintained in accordance with the principles of laboratory animal care under an Institutional Animal Care and Use Committee (IACUC) approved protocol.

## SUPPLEMENTARY MATERIALS



## References

[1] Bolden JE , Peart MJ , Johnstone RW . Anticancer activities of histone deacetylase inhibitors. Nat Rev Drug Discov. 2006; 5:769–84. 10.1038/nrd2133. 16955068

[2] Rosato RR , Almenara JA , Grant S . The histone deacetylase inhibitor MS-275 promotes differentiation or apoptosis in human leukemia cells through a process regulated by generation of reactive oxygen species and induction of p21CIP1/WAF1 1. Cancer Res. 2003; 63:3637–45. 12839953

[3] Vigushin DM , Coombes RC . Histone deacetylase inhibitors in cancer treatment. Anticancer Drugs. 2002; 13:1–13. 10.1097/00001813-200201000-00001. 11914636

[4] Burgess AJ , Pavey S , Warrener R , Hunter LJ , Piva TJ , Musgrove EA , Saunders N , Parsons PG , Gabrielli BG . Up-regulation of p21(WAF1/CIP1) by histone deacetylase inhibitors reduces their cytotoxicity. Mol Pharmacol. 2001; 60:828–37. 11562446

[5] Abbas T , Dutta A . p21 in cancer: intricate networks and multiple activities. Nat Rev Cancer. 2009; 9:400–14. 10.1038/nrc2657. 19440234 PMC2722839

[6] Liu S , Bishop WR , Liu M . Differential effects of cell cycle regulatory protein p21(WAF1/Cip1) on apoptosis and sensitivity to cancer chemotherapy. Drug Resist Updat. 2003; 6:183–95. 10.1016/S1368-7646(03)00044-X. 12962684

[7] Boder E . Ataxia-telangiectasia: some historic, clinical and pathologic observations. Birth Defects Orig Artic Ser. 1975; 11:255–70. 1096982

[8] Ju R , Muller MT . Histone deacetylase inhibitors activate p21(WAF1) expression via ATM. Cancer Res. 2003; 63:2891–7. 12782595

[9] Archer SY , Meng S , Shei A , Hodin RA . p21(WAF1) is required for butyrate-mediated growth inhibition of human colon cancer cells. Proc Natl Acad Sci U S A. 1998; 95:6791–6. 10.1073/pnas.95.12.6791. 9618491 PMC22637

[10] Saito A , Yamashita T , Mariko Y , Nosaka Y , Tsuchiya K , Ando T , Suzuki T , Tsuruo T , Nakanishi O . A synthetic inhibitor of histone deacetylase, MS-27-275, with marked *in vivo* antitumor activity against human tumors. Proc Natl Acad Sci U S A. 1999; 96:4592–7. 10200307 10.1073/pnas.96.8.4592 PMC16377

[11] Zhu W , Abbas T , Dutta A . DNA replication and genomic instability. Adv Exp Med Biol. 2005; 570:249–79. 10.1007/1-4020-3764-3_9. 18727504

[12] Nakano K , Mizuno T , Sowa Y , Orita T , Yoshino T , Okuyama Y , Fujita T , Ohtani-Fujita N , Matsukawa Y , Tokino T , Yamagishi H , Oka T , Nomura H , Sakai T . Butyrate activates the WAF1/Cip1 gene promoter through Sp1 sites in a p53-negative human colon cancer cell line. J Biol Chem. 1997; 272:22199–206. 10.1074/jbc.272.35.22199. 9268365

[13] Xiao H , Hasegawa T , Isobe K . Both Sp1 and Sp3 are responsible for p21waf1 promoter activity induced by histone deacetylase inhibitor in NIH3T3 cells. J Cell Biochem. 1999; 73:291–302. 10321829

[14] Beishline K , Azizkhan-Clifford J . Sp1 and the 'hallmarks of cancer'. FEBS J. 2015; 282:224–58. 10.1111/febs.13148. 25393971

[15] Hendzel MJ , Wei Y , Mancini MA , Van Hooser A , Ranalli T , Brinkley BR , Bazett-Jones DP , Allis CD . Mitosis-specific phosphorylation of histone H3 initiates primarily within pericentromeric heterochromatin during G2 and spreads in an ordered fashion coincident with mitotic chromosome condensation. Chromosoma. 1997; 106:348–60. 10.1007/s004120050256. 9362543

[16] Wei Y , Mizzen CA , Cook RG , Gorovsky MA , Allis CD . Phosphorylation of histone H3 at serine 10 is correlated with chromosome condensation during mitosis and meiosis in Tetrahymena. Proc Natl Acad Sci U S A. 1998; 95:7480–4. 10.1073/pnas.95.13.7480. 9636175 PMC22657

[17] Gautier J , Solomon MJ , Booher RN , Bazan JF , Kirschner MW . cdc25 is a specific tyrosine phosphatase that directly activates p34cdc2. Cell. 1991; 67:197–211. 10.1016/0092-8674(91)90583-k. 1913817

[18] Warrener R , Beamish H , Burgess A , Waterhouse NJ , Giles N , Fairlie D , Gabrielli B . Tumor cell-selective cytotoxicity by targeting cell cycle checkpoints. FASEB J. 2003; 17:1550–2. 10.1096/fj.02-1003fje. 12824307

[19] Gartel AL , Serfas MS , Tyner AL . p21--negative regulator of the cell cycle. Proc Soc Exp Biol Med. 1996; 213:138–49. 10.3181/00379727-213-44046. 8931660

[20] Schmidt M , Fan Z . Protection against chemotherapy-induced cytotoxicity by cyclin-dependent kinase inhibitors (CKI) in CKI-responsive cells compared with CKI-unresponsive cells. Oncogene. 2001; 20:6164–71. 10.1038/sj.onc.1204814. 11593424

[21] Ferrandiz N , Caraballo JM , Albajar M , Gomez-Casares MT , Lopez-Jorge CE , Blanco R , Delgado MD , Leon J . p21(Cip1) confers resistance to imatinib in human chronic myeloid leukemia cells. Cancer Lett. 2010; 292:133–9. 10.1016/j.canlet.2009.11.017. 20042273

[22] Koster R , di Pietro A , Timmer-Bosscha H , Gibcus JH , van den Berg A , Suurmeijer AJ , Bischoff R , Gietema JA , de Jong S . Cytoplasmic p21 expression levels determine cisplatin resistance in human testicular cancer. J Clin Invest. 2010; 120:3594–605. 10.1172/JCI41939. 20811155 PMC2947220

[23] Boultwood J . Ataxia telangiectasia gene mutations in leukaemia and lymphoma. J Clin Pathol. 2001; 54:512–6. 10.1136/jcp.54.7.512. 11429421 PMC1731462

[24] Thompson LH , Schild D . Recombinational DNA repair and human disease. Mutat Res. 2002; 509:49–78. 10.1016/s0027-5107(02)00224-5. 12427531

[25] Luchenko VL , Litman T , Chakraborty AR , Heffner A , Devor C , Wilkerson J , Stein W , Robey RW , Bangiolo L , Levens D , Bates SE . Histone deacetylase inhibitor-mediated cell death is distinct from its global effect on chromatin. Mol Oncol. 2014; 8:1379–92. 10.1016/j.molonc.2014.05.001. 24954856 PMC4646083

[26] Yang C , Tang X , Guo X , Niikura Y , Kitagawa K , Cui K , Wong ST , Fu L , Xu B . Aurora-B mediated ATM serine 1403 phosphorylation is required for mitotic ATM activation and the spindle checkpoint. Mol Cell. 2011; 44:597–608. 10.1016/j.molcel.2011.09.016. 22099307 PMC3228519

[27] Marchi E , Paoluzzi L , Scotto L , Seshan VE , Zain JM , Zinzani PL , O’Connor OA . Pralatrexate is synergistic with the proteasome inhibitor bortezomib in *in vitro* and *in vivo* models of T-cell lymphoid malignancies. Clin Cancer Res. 2010; 16:3648–58. 10.1158/1078-0432.CCR-10-0671. 20501616

[28] Paoluzzi L , Scotto L , Marchi E , Zain J , Seshan VE , O’Connor OA . Romidepsin and belinostat synergize the antineoplastic effect of bortezomib in mantle cell lymphoma. Clin Cancer Res. 2010; 16:554–65. 10.1158/1078-0432.CCR-09-1937. 20068080

[29] Zhao L , Wientjes MG , Au JL . Evaluation of combination chemotherapy: integration of nonlinear regression, curve shift, isobologram, and combination index analyses. Clin Cancer Res. 2004; 10:7994–8004. 10.1158/1078-0432.CCR-04-1087. 15585635

