# Supplementary material for: ATM inhibition overcomes resistance to histone deacetylase inhibitor due to p21 induction and cell cycle arrest
Source: Oncotarget. 2020 Sep 15;11(37):3432–42. doi: 10.18632/oncotarget.27723 (PMC7500109; doi:10.18632/oncotarget.27723)
Supplement: Supplementary file 1 [file oncotarget-11-3432-s001.pdf]

# ATM inhibition overcomes resistance to histone deacetylase inhibitor due to p21 induction and cell cycle arrest

## SUPPLEMENTARY MATERIALS

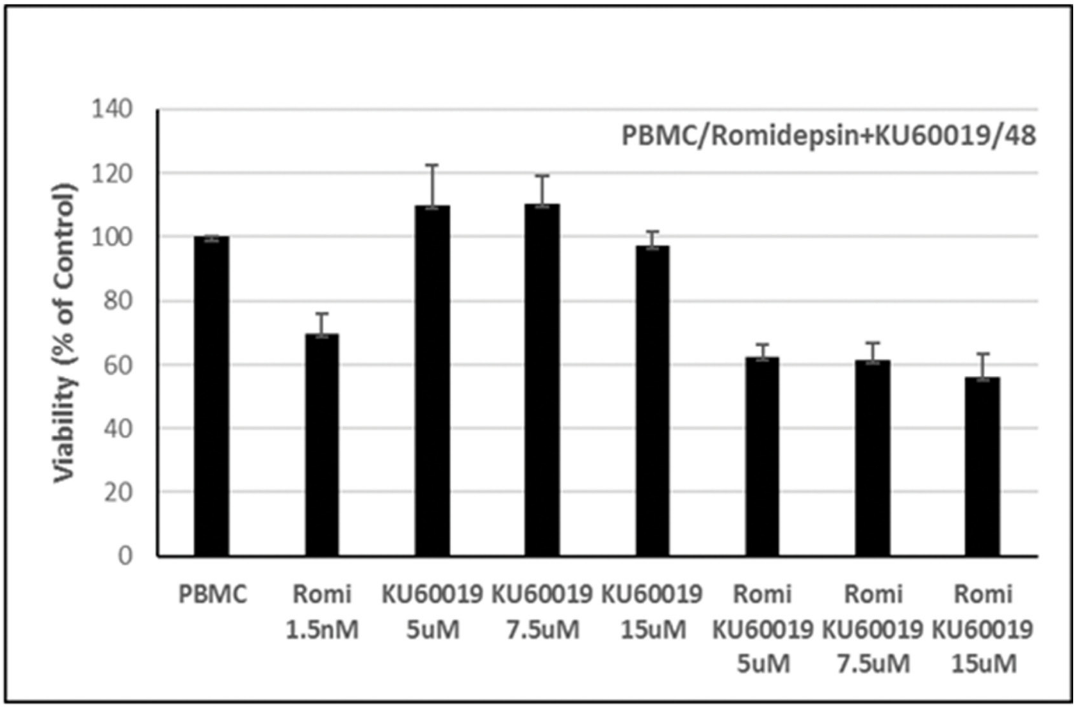


| Time | 24h | 48h | 72h |
|---|---|---|---|
| KU60019 5.0 uM Romidepsin 1.5 nM | 0.89 | 1.25 | 0.79 |
| KU60019 7.5 uM Romidepsin 1.5 nM | 0.98 | 1.25 | 1.54 |
| KU60019 15 uM Romidepsin 1.5 nM | 102 | 0.82 | 1.08 |

**Supplementary Figure 1: The ATM inhibitor KU60019 and Romidepsin lower toxicity effect in healthy PBMC.** Cytotoxicity effects observed at 48 hours of exposure to single agents and combination for PBMC isolated from a healthy patient. Also shown are RRR values at 24, 48, and 72 hours of exposure to romidepsin in combination with KU60019. Error bars represent SD.

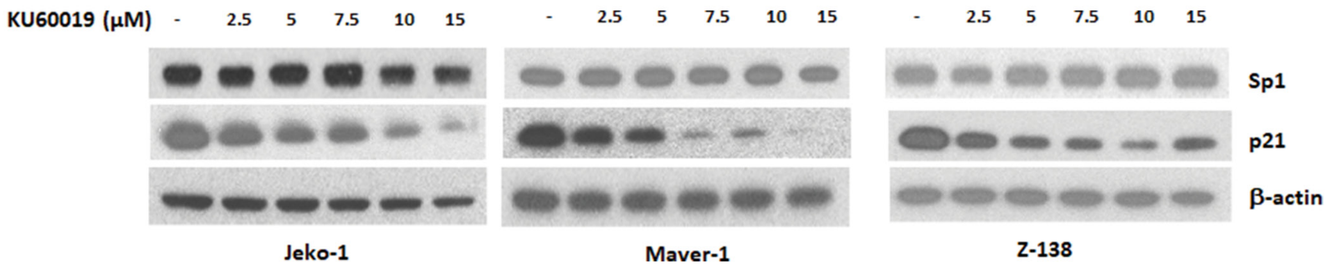


**Supplementary Figure 2: The ATM inhibitor KU60019 decreases p21 expression in a concentration dependent manner.** Western blot analysis of Sp1 and p21 protein levels in Jeko-1, Maver-1, and Z-138 cells after 24 hours exposure to increased concentration of KU60019.

## Supplementary Table 1: Primers for Sp1, Sp3, p21 and β-actin RT-PCR expression analysis

| Gene | Forward primer | Reverse primer | Amplicon length (bp) |
|---|---|---|---|
| SP1 | AGGCTGTGGGAAAGTGTATG | GGTAATAAGGGCTGAAGGAGTG | 343 |
| SP3 | TGGAGCCTTCACTTCAACTC | CTTCTTCATCTACCACCTGTACTC | 245 |
| p21 | CTGTCACTGTCTTGTACCCTTG | GATGTAGAGCGGGCCTTTG | 220 |
| β-Actin | TCACCCACACTGTGCCCATCTACGA | CAGCGGAACCGCTCATTGCCAATGG | 295 |
